# Assessment of *Auricularia cornea* var. Li. polysaccharides potential to improve hepatic, antioxidation and intestinal microecology in rats with non-alcoholic fatty liver disease

**DOI:** 10.3389/fnut.2023.1161537

**Published:** 2023-06-12

**Authors:** Tiantian Zhou, Xue Mao, Wei Jiang, Yu Pan, Xijun Chen, Jihua Hu, Xianghui Kong, Haihua Xia

**Affiliations:** ^1^Institute of Microbiology, Heilongjiang Academy of Sciences, Harbin, China; ^2^Key Laboratory of Flexible Electronics, Institute of Advanced Materials, Nanjing Tech University, Nanjing, China

**Keywords:** *Auricularia cornea* var. Li., polysaccharides, high-fat diet, non-alcoholic fatty liver disease, gut microbiota

## Abstract

Non-alcoholic fatty acid liver disease (NAFLD) is a reputed global health concern, affecting children and young adults. Accumulating evidence suggests that edible fungi polysaccharides have the potential to relieve NAFLD. Our previous study found that *Auricularia cornea* var. Li. polysaccharides (ACP) could improve immune by regulating gut microbiota. However, its NAFLD-alleviating potentials have been scarcely reported. This study analyzed the protective effects of *Auricularia cornea* var. Li. polysaccharides on high-fat diet (HFD)-induced NAFLD and mechanistic actions. We first analyzed the histology and hepatic lipid profile of animals to evaluate this variant’s ameliorating effects on NAFLD. Then, antioxidant and anti-inflammatory potentials of ACP were studied. Finally, we explored changes in the gut microbiome diversity for mechanistic insights from the gut-liver region. Results showed that supplementation with ACP substantially reduced homeostasis model assessment-insulin resistance (HOMA-IR), body fat, liver index rates and weight gain (*p* < 0.05). This variant also improved HDL-C levels while decreasing triglyceride (TG), total cholesterol (TC), and low-density lipoprotein cholesterol (LDL-C) levels which were initially triggered by HFD. ACP mediation also decreased the serum alanine aminotransferase (ALT) and aspartate aminotransferase (AST) levels considerably with H&E technique indicating that it can reduce liver lipid accumulation, thus lowering liver damages risks (*p* < 0.05). The antioxidant potentials of ACP were also demonstrated as it decreased the hepatic levels of malondialdehyde (MDA) and increased the activities of superoxide dismutase (SOD), catalase (CAT), and glutathione peroxidase (GSH-PX). Proinflammatory markers like IL-6, IL-1β and TNF-α concentrations were decreased by ACP supplementation, accompanied with increased IL-4 levels. Finally, ACP supplementation regulated the intestinal microbiota to near normal patterns. In all, ACP protects HFD-induced NAFLD by improving liver characteristics and regulating colonic flora composition, our findings assert that ACP can be a promising strategy in NAFLD therapy.

## Introduction

Nonalcoholic fatty liver disease (NAFLD), is a highly pervasive liver condition globally (25.24% incidence rate), common among young adults and children (1). In China, it is only second to viral hepatitis in chronic liver disease causes (2). Although, the mechanisms regulating its pathogenesis is still unclear, NAFLD symptoms range from simple steatosis to severe inflammation, with the possibility of cirrhosis (3). Following the ‘multiple hit’ hypothesis, the gut microbiome could be linked to the onset and development of NAFLD (4). Current NAFLD treatments such as medicinal intervention and bariatric surgery have several side effects, including increased weight and osteoporosis (5), raising the need to explore alternative methods for NAFLD alleviation.

Notable biosafety and industrial importance have been identified in polysaccharides from edible fungi (6). Moreso, it has been reported previously that polysaccharides could have immunomodulatory and antioxidant activities. Findings suggested that *Auricularia polytricha* aqueous extract supplementation showed protective material against NAFLD by attenuating inflammatory response, oxidative stress and lipid deposition (7). It was found that polysaccharides from *Auricularia auricula* and *Auricularia polytricha* could inhibit oxidative stress, nuclear factor kappa-B (NF-κB) signaling and proinflammatory cytokine production (8). It has been reported that *Auricularia auricula* polysaccharides demonstrated the regulatory effects of endogenous metabolism and gut microbiota composition (9). The results of showed that the *Auricularia auricula* polysaccharide interventions had a potential to improve hyperlipidemia with the modulation of gut microbiota structure (10). Recent studies have shown that dietary supplementation of *Auricularia auricula-judae* polysaccharides could alleviate nutritional obesity in rats via regulating inflammatory response and lipid metabolism (11). It is thus possible that *Auricularia* polysaccharides could be useful in NAFLD prevention and treatment.

A genetically-stable variant of *Auricularia cornea* species known as *Auricularia cornea* var. Li could be produced in large amounts in China (12). Through several pathways, Wang et al. reported that this variant exerted hepatoprotective effects against the alcoholic liver diseases (13). *Auricularia cornea* var. Li. polysaccharides also has good antioxidant capacity, antinephritic effects, unique phenotype and potential medicinal properties (14). Our previous study found that *Auricularia cornea* var. Li. polysaccharides supplementation could improve immune by regulating gut microbiota (15). However, little is known about its NAFLD-alleviating potentials.

The objective of this work to analyze the protective effects of *Auricularia cornea* var. Li. polysaccharides on HFD-induced NAFLD and its related mechanism. The ameliorative effects of *Auricularia cornea* var. Li. polysaccharides on hepatic lipid profile and histological alterations, and inflammation cytokines in immunosuppressed rats were studied. In addition, the regulatory effect of *Auricularia cornea* var. Li. polysaccharides on the gut microbiota was analyzed. These findings will give further understanding regarding NAFLD pathogenesis and a framework exploring the possible application of *Auricularia cornea* var. Li. Polysaccharides in NAFLD therapy.

## Materials and methods

### Isolation and purification of *Auricularia cornea* var. Li. Polysaccharides

*Auricularia cornea* var. Li. was purchased from the edible fungus base in Heilongjiang province. The extraction methods of AAP are described by (16). Make a few changes. In short, first extract 20 g auriculae powder with 1 L deionized water at 90°C for 2 h. The total extract was concentrated to 1/3 of its original volume under reduced pressure and precipitated overnight with three-volume frozen 95% ethanol at 4°C. The protein content of precipitated solution was removed by Sevag method. The aqueous solution was dialyzed with deionized water for 3 days (7 kD), and the water was changed 3 times a day. Crude polysaccharides are obtained by concentration and freeze-drying. Purification was performed using anion exchange columns to separate the components of the crude polysaccharide, followed by further purification using gel columns and partial lyophilization of the final polysaccharide for further study.

### Animals and experimental design

Thirty-six specific-pathogen-free (SPF) male Sprague–Dawley rats (160 ~ 180 g) were supplied by the Shanghai Slack Laboratory Animal Co. LTD [Shanghai, China, SCXK (Shanghai) 2012–0002]. All feeding was done in an animal room (22 ± 0.5°C and 55 ± 5%) with 12 h light/12 h dark cycles. Chow and water were made available *ad libitum* for the one-week acclimatization period. Afterward, rats were placed in three groups (*n* = 12 rats per group), including the control, HFD and *Auricularia cornea* var. Li. polysaccharides (ACP) groups. Rats in the control groups were fed with a regular diet throughout the experiment, while HFD group and ACP group fed with a HFD for 6 weeks. ACP (200 mg kg^−1^) solution was orally administrated to the rats in the ACP group, while equal volume of distilled water were administrated to the rats in the HFD group. Feed formulas are reported in Supplementary Table S1. Rats were humanely sacrificed after a 12 h fasting period. The blood was placed on ice for 2 h, and then the serum was obtained by centrifugation. The livers, epididymal adipose tissue and perirenal adipose tissue of rats were weighed and recorded, then stored at −80°C. The blood and small intestine were obtained for next experiments. The Ethics Committee of the First Affiliated Hospital of Heilongjiang University of Chinese Medicine approved all animal experiments and protocols (2022060801). Liver index and Body fat rate were calculated using the following formula:


Liver index(%)=liver weight(g)/body weight(g)×100%.

$$\begin{array}{l} \mathrm{Body}\;\mathrm{fat}\;\mathrm{rate}\;\left(\%\right)\\ \;\;=\left[\mathrm{epididymal adipose tissue}\;\left(g\right)+\mathrm{perirenal adipose tissue}\;\left(g\right)\right]\\ \;\;/\mathrm{body weight}\;\left(g\right)\times 100\%. \end{array}$$

### Liver histopathological analysis

Referring to the method of (17) with slight modifications. We placed all liver tissues in paraffin wax and made thin slices 5 μm thick，and then stained with hematoxylin and eosin (H&E). Histology of these sections were observed using a light microscope (Nikon E100, 200 × magnification).

### Determination of TC, TG, LDL-C, and HDL-C in the liver

The liver tissues were homogenized in aseptic PBS (1: 9, w/v), succeeded by centrifugation at 10,000 r/min for 15 min at 4°C. The concentration of TC, TG, HDL-C, and LDL-C was detected using Rats ELISA kits (Roche Diagnostics GmbH, Mannheim, German) based on the instructions of the manufacturer.

### Serum ALT, AST, FBG, and FINs determination

Serum ALT, AST, fasting plasma glucose (FBG)and fasting insulin (FINs) levels were obtained using mouse kits (Conodi Creatures, Fujian, China) by following the manufacturer’s directives. HOMA-IR was calculated using the following formula:


HOMA−IR=FBG(mmol/L)×FINS(μU/mL)/22.5.

### Determination of the level of oxidative stress in the liver

Liver oxidative stress in the different groups were assessed by measuring malondialdehyde (MDA), superoxide dismutase (SOD), catalase (CAT), and glutathione peroxidase (GSH-PX) levels using commercial kits obtained from Jiancheng Creatures Co Ltd., (Nanjing, China).

### Inflammatory cytokines measurement

Aseptic PBS (1:9 w/v) was used to homogenize liver tissues and centrifuged at 10,000 r/min for 10 min at 4°C. Afterward, the TNF-α, IL-6, IL-4 and IL-1β were detected using ELISA kits obtained from Conodi Creatures Co Ltd., (Fujian, China) by following the manufacturer’s instructions.

### DNA extraction and 16S rRNA gene sequencing

Total bacterial genomic DNA was isolated from fecal samples using the Fast DNA SPIN extraction kits (MP Biomedicals, Santa Ana, CA, United States) according to the manufacturer’s instructions. 0.8% agarose gel electrophoresis was used to determine the DNA molecular size, and NanoDrop NC-2000 Spectrophotometer was used to quantify DNA. Afterwards, the bacterial 16S rRNA genes V3–V4 region was amplified by polymerase chain reaction (PCR) with the forward primer 338F (5’-ACTCCTACGGGAGGCAGCA-3′) and the reverse primer 806R (5’-GGACTACHVGGGTWTCTAAT-3′). PCR amplicons were purified with Agencourt AMPure Beads (Beckman Coulter, Indianapolis, IN) and quantified using the PicoGreen dsDNA Assay Kit (Invitrogen, Carlsbad, CA, United States). The TruSeq Nano DNA LT Library Prep Kit (Illumina, United States) was used for library construction. Sequencing was performed using the MiSeq Reagent Kit v3 (Shanghai Personal Biotechnology Co., Ltd.,Shanghai, China) on the Illumina MiSeq platform. Following data unloading, we sieved out low-quality reads (reads containing N bases, 3 ‘end adapter, reads with average quality value ≥20). Data obtained by pair-end sequencing was spliced into long tags using FastQ-Join software. Spliced tags were carefully filtered to obtain effective data (chimeras were also removed as described by (18)). Finally, the QIME software (v1.9.1) and UcLUST method were used to cluster OTU units (19) and align the representative sequence (20, 21).

### Statistical analysis

All data were analyzed using SPSS 22.0 software (SPSS Inc., Chicago, IL, United States). Statistical analysis of Duncan’s multiple range tests after one-way analysis of variance (ANOVA). For all analyses, at *p* < 0.05, the differences were considered significant.

## Results

### Effects of ACP supplementation on body weight, liver index, body fat rate, and HOMA-IR of HFD-Fed rats

**Figure 1:**
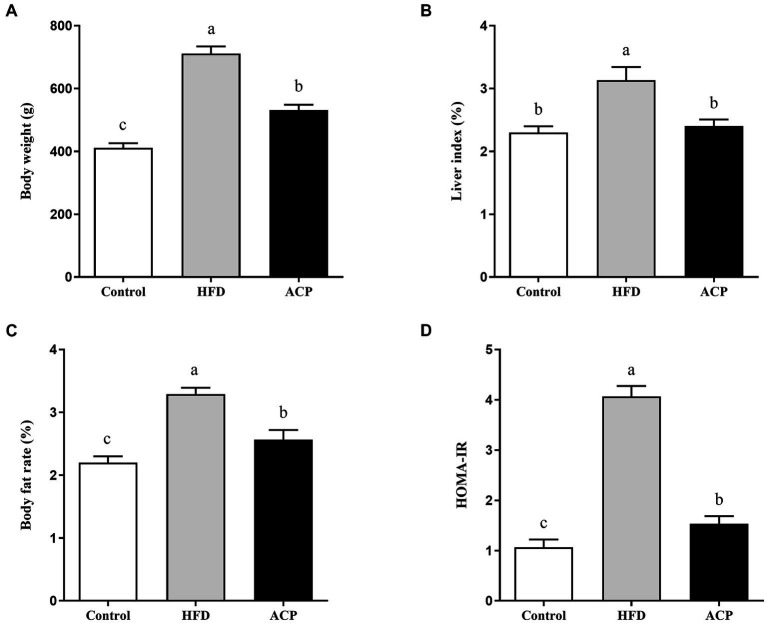
Effect of ACP supplementation on body weight **(A)**, liver index **(B)**, body fat rate **(C)** and **(D)** HOMA-IR of HFD-fed rats. Control, control group; HFD, high fat diet group; and ACP, *A*. *cornea* var. Li. polysaccharides group. Values are expressed as mean ± SD (*n* = 12). Different superscript letters indicate significant differences (*p* < 0.05).

In Figure 1A, we observed that HFD substantially increased the final body weight, which was reversed in the ACP supplementation group. Liver index analysis results indicate that HFD induction gave a marked increase in the measured parameters which were reversed after ACP supplementation (Figure 1B). As expected, the HFD group had heightened body fat rates compared to the control group (*p* < 0.05), which was again ameliorated in the polysaccharide group (Figure 1C). The HFD group also showed increased HOMA-IR levels (*p* < 0.05) which was reversed to control group levels after ACP supplementation (Figure 1D). In conclusion, the addition of ACP normalized the increase in body weight, liver index, and body fat percentage in HFD-fed rats. At the same time, HOMA-IR was reduced.

### Effects of ACP supplementation on lipid accumulation in the liver

**Figure 2:**
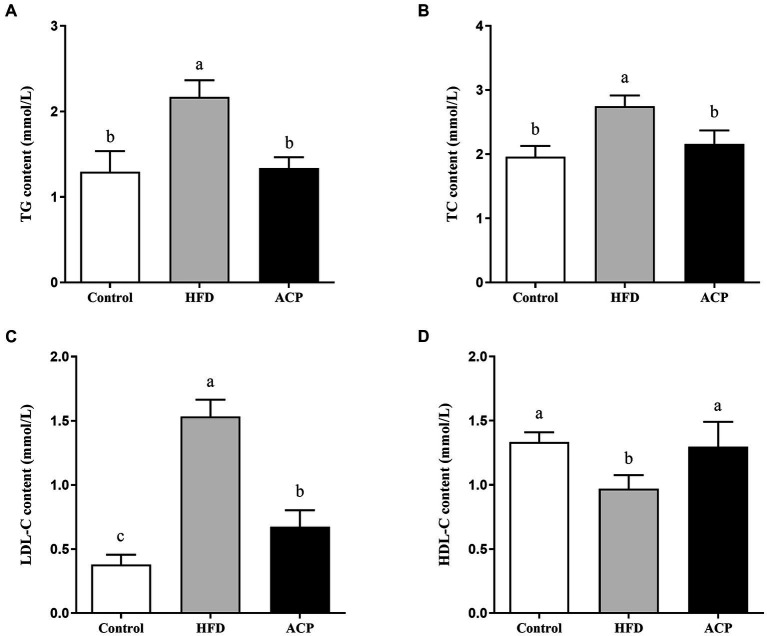
Effects of ACP supplementation on lipid accumulation in the liver in HFD-fed rats. Control, control group; HFD, high fat diet group; and ACP, *A*. *cornea* var. Li. polysaccharides group. **(A)** Hepatic triglyceride (TG) level. **(B)** Hepatic total cholesterol (TC) level. **(C)** Hepatic low density lipoprotein cholesterol (HDL-C) level. **(D)** Hepatic high density lipoprotein cholesterol (LDL-C) level. Values are expressed as mean ± SD (*n* = 12). Different superscript letters indicate significant differences (*p* < 0.05).

We measured the liver TC, TG, LDL-C, and HDL-C using the ELISA kit. The HFD group showed elevated TG, TC, and LDL-C levels compared to the control group (*p* < 0.05) (Figures 2A–C). Moreover, the HDL-C levels were lowered (Figure 2D). Compared to the HFD group, LDL-C, TC and TG levels were significantly lower when ACP was administered (*p* < 0.05), while HDL-CS levels were significantly increased (*p* < 0.05), returning to similar levels in the control group.

### Effects of ACP supplementation on ALT and AST levels in the serum

**Figure 3:**
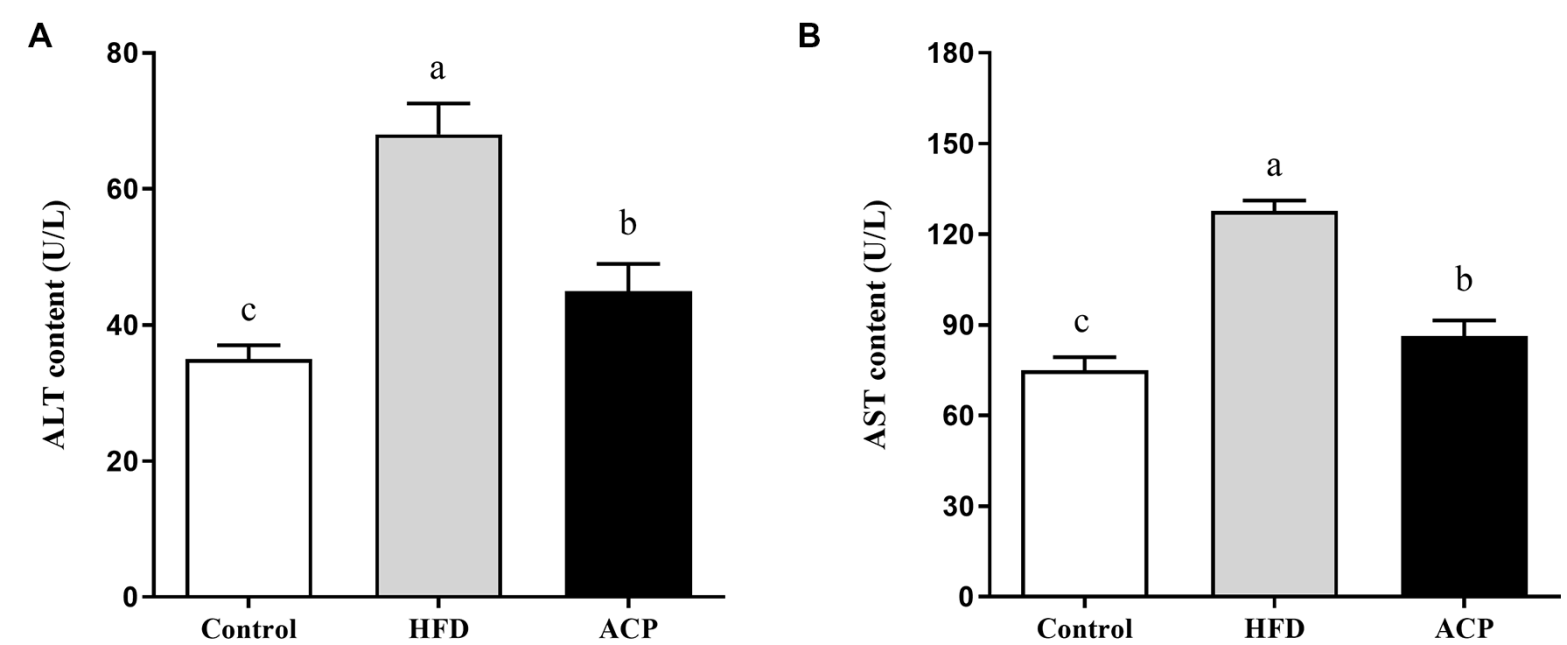
Effects of ACP supplementation on liver function. Control, control group; HFD, high fat diet group; and ACP, *A*. *cornea* var. Li. polysaccharides group. **(A)** Serum alanine aminotransferase (ALT) level. **(B)** Serum aspartate aminotransferase (AST) level. Values are expressed as mean ± SD (*n* = 12). Different superscript letters indicate significant differences (*p* < 0.05).

We used the ELISA technique to assess liver function by evaluating the ALT and AST levels of rats. ALT and AST activities were significantly increased in the HFD group compared to the control group (*p* < 0.05) (Figures 3A,B). The increase in HFD-induced ALT and AST activity was significantly suppressed in the ACP supplemented group compared to the HFD group (*p* < 0.05).

### Effects of ACP supplementation on histological alterations of the liver tissues

**Figure 4:**
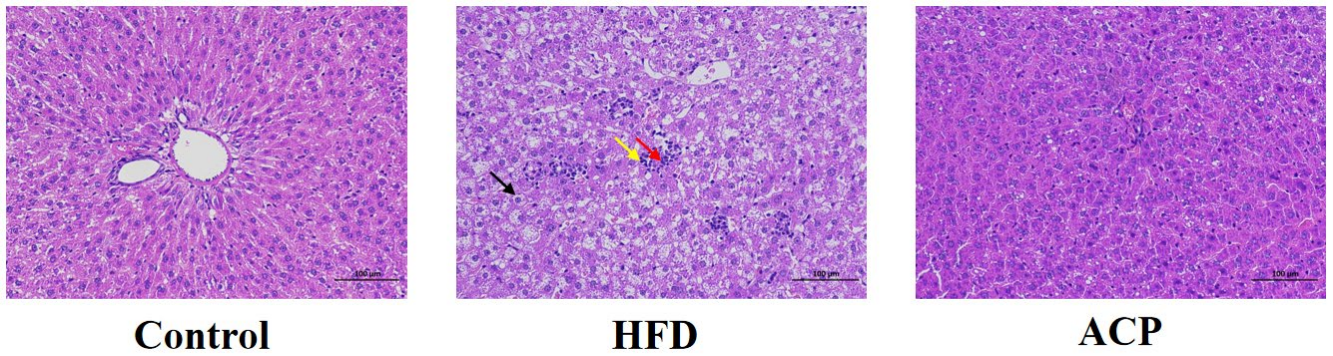
Effects of ACP supplementation on histological alterations of liver. Control, control group; HFD, high fat diet group; and ACP, *A*. *cornea* var. Li. polysaccharides group. Values are expressed as mean ± SD (*n* = 12). Different superscript letters indicate significant differences (*p* < 0.05).

As shown in Figure 4, in the Control group, the structure of the hepatic lobule was clear, the hepatic cords was arranged neatly, no obvious expansion or compression of the hepatic sinuses was observed, and no obvious inflammation was observed. A large number of hepatocyte balloon degeneration, cell swelling, nuclei centered, cytoplasmic vacuolation (black arrow), multiple focal lymphocyte infiltration (yellow arrow), rare hepatocyte necrosis, and nuclear fragmentation (red arrow) were found in the tissue of HFD-fed rats. However, administration of ACP was effective in reversing this HFD-induced trend, suggesting that ACP acts as a positive hepatoprotective agent.

### Effects of ACP supplementation on the oxidative stress

**Figure 5:**
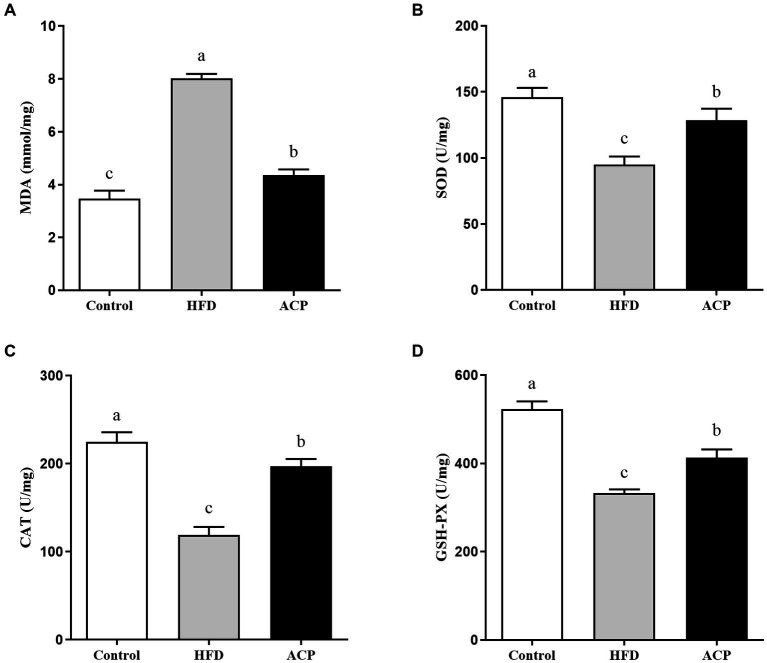
Effects of ACP supplementation on the oxidative stress. Control, control group; HFD, high fat diet group; and ACP, *A*. *cornea* var. Li. polysaccharides group. **(A)** MDA, **(B)** SOD, **(C)** CAT, and **(D)** GSH-PX. Values are expressed as mean ± SD (*n* = 12). Different superscript letters indicate significant differences (*p* < 0.05).

In this study, we measured selected oxidative stress indicators to evaluate the effect of ACP supplementation on NAFLD. As presented in Figure 5, compared with the control group, the rats in the HFD group showed higher MDA levels (*p* < 0.05), with much reduced SOD, CAT, and GSH-PX in the liver tissues (*p* < 0.05). However, MDA levels were significantly lower in rats receiving ACP intervention compared to the HFD group (*p* < 0.05). Meanwhile, the levels of SOD, CAT, and GSH-PXs were significantly increased (*p* < 0.05). All these results suggest that ACP supplementation can effectively inhibit HFD-induced oxidative stress damage in NAFLD rats.

### Effects of ACP supplementation on cytokine contents in the liver

**Figure 6:**
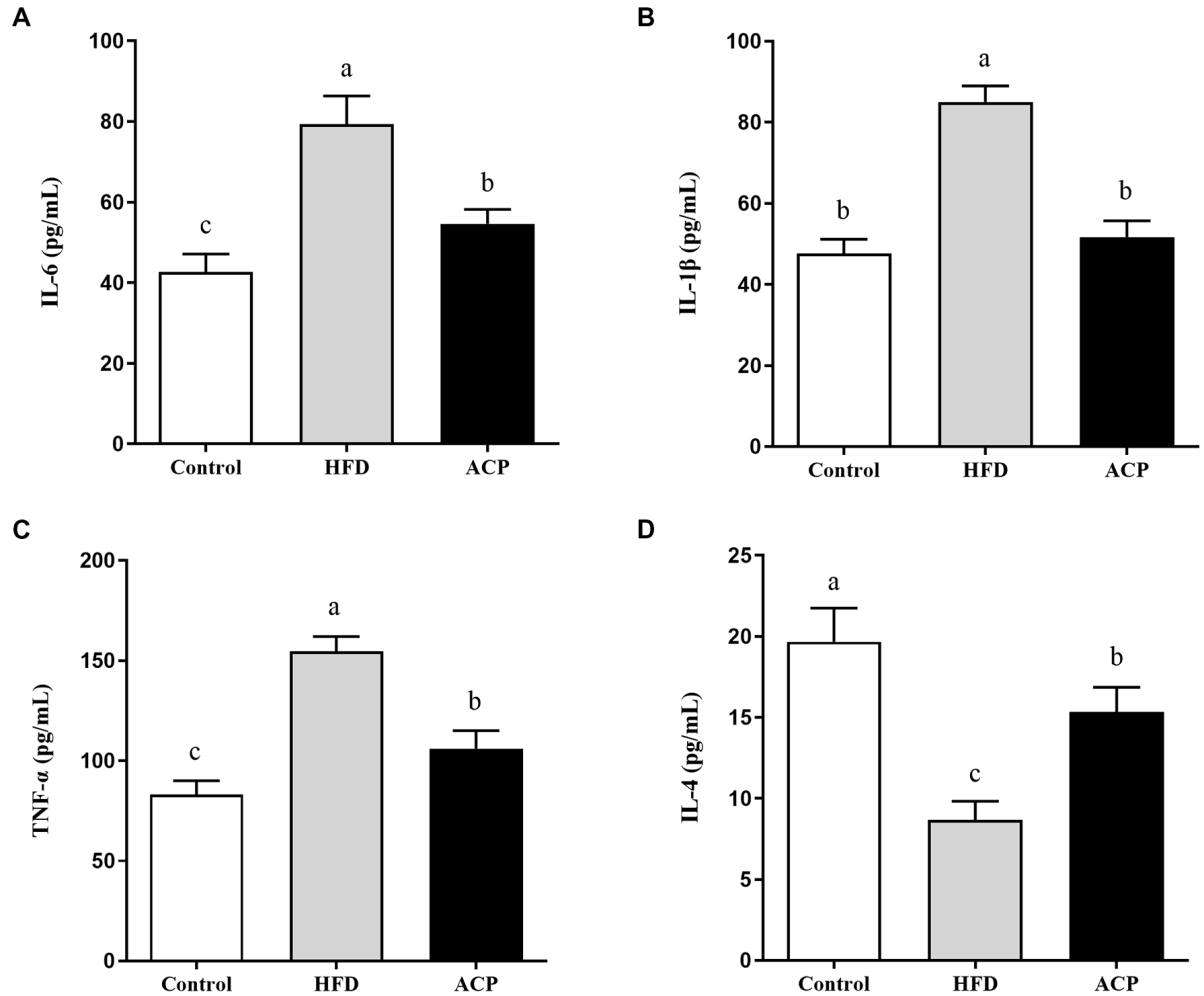
Effects of ACP supplementation on cytokine contents. Control, control group; HFD, high fat diet group; and ACP, *A*. *cornea* var. Li. polysaccharides group. **(A)** IL-6; **(B)** IL-1β; **(C)** TNF-α; and **(D)** IL-4. Values are expressed as mean ± SD (*n* = 12). Different superscript letters indicate significant differences (*p* < 0.05).

Long-term use of HFD can cause persistent low-grade inflammation and contribute to NAFLD. Following analyses of selected cytokines, our results (Figure 6) showed that HFD rats exhibited heightened levels of IL-6, IL-1β and TNF-α concentrations compared to the control group (*p* < 0.05). Furthermore, IL-4 levels decreased. Compared with the HFD group, IL-6, IL-1β and TNF-α levels were significantly lower and IL-4 levels were significantly higher after ACP supplementation (*p* < 0.05), converging to the control group.

### Effects of ACP supplementation on gut microbiota

**Figure 7:**
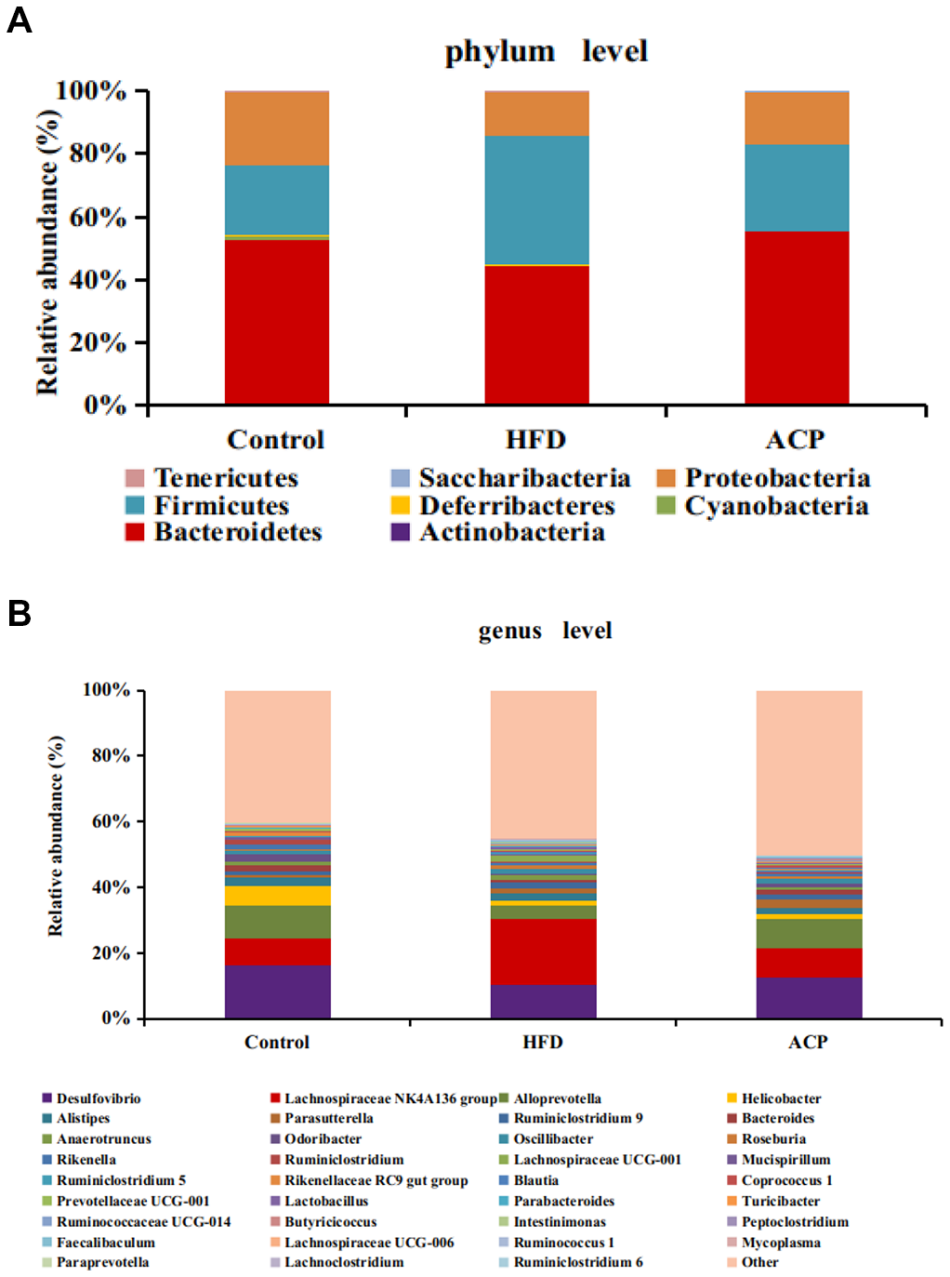
Changes of the gut microbiota at the phylum **(A)** and genus **(B)** level after ACP supplementation. Control, control group; HFD, high fat diet group; and ACP, *A*. *cornea* var. Li. polysaccharides group.

To investigate whether mixed lactobacilli have an important role in the bacterial communities of NAFLD-induced rats, the gut microbiota of rats was analyzed by sequencing the 16S rDNA variable region V3–V4. The phylum levels of the gut microbiome samples are shown as Figure 7A. Firmicutes and Bacteroidota were the dominant phyla with a total abundance of more than 70%, followed by Proteobacteria and Deferribacteres. The relative abundance of Firmicutes was increased and the relative abundance of Bacteroidota were decreased in the HFD group. In reversing this trend, ACP supplementation lowered Firmicutes abundance level, raised Bacteroidetes diversity gave a decreased ratio of Firmicutes: Bacteroidetes. The genus levels were shown in Figure 7B. In the HFD group, the relative abundances of *Bifidobacterium*, *Bacteroides*, *Odoribacter*, *Alloprevotella*, *Rikenellaceae RC9 gut group* and *Blautia* were decreased compared with the control group. In contrast, the abundance of these genuses in the ACP supplementation were increased in the compare with the HFD group. Moreover, the relative abundances of *Parabacteroides*, *Lachnoclostridium*, *Lachnospiraceae NK4A136 group* and *Roseburia* were increased, which was decreased when compared to the HFD group.

## Discussion

The pervasive effects of NAFLD on a global scale is a public health concern that has warranted continued research into its mitigation (22). Using environment-friendly approaches such as edible fungus that secrete biologically-active compounds can be a promising technique for its therapy (23, 24). Our previous study found that polysaccharides supplementation with *Auricularia cornea* var. Li. (ACP) could improve immune by regulating gut microbiota (15), but its role in alleviating NAFLD has been poorly studied. The objective of this work to analyze the protective effects of *Auricularia cornea* var. Li. polysaccharides on HFD-induced NAFLD and its related mechanism. In this study, HFD significantly increased the body weight gain, liver index, body fat rate and HOMA-IR, but this was restored to normalcy by supplementation with ACP, in agreement with Naudrin et al. (25), who opined that supplementation with this polysaccharide could be protective against NAFLD induced by HFD. It is known that HFD-induced NAFLD is featured by increased TC, TG, and LDL levels and decreased HDL levels (26). Interestingly, it has also been reported that when these NAFLD biomarker levels are lowered, the condition is reversed (27, 28). We observed that the hepatic levels of TC, TG, and LDL and lowered the HDL levels were induced by HFD. This trend was reversed by supplementation with ACP, which was in line with the effects of polysaccharides from *Enteromorpha prolifera* in previous study (29). HFD also caused liver injury *via* significant fat accumulation, but this was lowered after ACP administration in NAFLD rats. Lipid deposition and toxicity levels which indicate inflammations and liver injury, are typically measured by serum AST and ALT levels (30). In our study, the HFD rats group should considerably high serum AST and ALT levels, which was reversed by ACP supplementation. These indicate that mediation with this polysaccharide could attenuate HFD-induced liver injury and protect liver function. The biomarkers used to measure liver oxidative stress (lipid peroxidation) include MDA, SOD, CAT and T-AOC. SOD breaks down superoxide anion, which is required as a catalyst in reducing O_2_^▪−^ to H_2_O_2_, this is then converted to water by CAT (31). MDA can be a reliable *in vivo* biomarker of oxidative stress (32). Zhu et al. reported that chicory polysaccharide could improve the NAFLD in a rat model (33). Our results showed that ACP supplementation significantly decreased the hepatic levels of MDA and increased the activities of SOD, CAT, and GSH-PX, indicating that ACP supplementation could alleviate the NAFLD through elevating antioxidant capacity. In addition to immune response regulations, cytokines also play a role in repair of damaged tissues. Microbes in the intestine are known to directly or indirectly trigger immune responses and inflammations *via* cytokines like IL-6, IL-1β and TNF-α, with possible NAFLD-ameliorating effects (34). In this study, we observed that while IL-6, IL-1β and TNF-α levels were significantly raised by induced HFD conditions while IL-4 levels were lowered. This scenario was reversed by ACP mediation, suggesting that it could be strategic in NAFLD therapy *via* cytokine level regulation.

There is increasing evidence that NAFLD onset could be mitigated or aggravated by microbes in the gut (35). Our results showed that in the HFD group, the relative abundance of Bacteroidetes decreased substantially, noting an unusual increase in Firmicutes levels, aggreging with earlier studies that report high Firmicutes: Bacteroidetes ratio (36–38). The Firmicutes phylum have been reported to raise calories absorption which triggers obesity biomarkers with suppressed Bacteroidetes levels (39, 40). Following this scenario, ACP supplementation reversed this trend by raising Bacteroidetes abundance and also lowered the Firmicutes: Bacteroidetes ratio. Other reports demonstrated that the presence of *Bifidobacterium* (41), *Bacteroides* (42, 43), and *Alloprevotella* (44) were negatively correlated with obesity. As a non-pathogenic gut microbe, *Bifidobacterium* contributes to gut and intestinal health by secreting pathogen-inhibiting organic acids and lowers intestinal environment pH (45). *Blautia* is well-known butyrate producers, which is associated with a wide range of health benefits, including improved body composition and weight loss (46, 47). It has been reported that orally administered *Odoribacter laneus* could improve glucose control and inflammatory profile in obese rats by depleting circulating succinate (48). *Alloprevotella* belonging to the Bacteroidetes phylum has shown anti-inflammatory effects (49).

LPS is secreted by abundances of *Parabacteroides* (50, 51), *Lachnoclostridium* (52), *Lachnospiraceae NK4A136 group* (53) and *Romboutsia* (51), triggering proinflammatory cytokines, obesity, and insulin resistance. Again, organic acid-producing bacteria can be substantially suppressed by *Lachnoclostridium*, *Lachnospiraceae NK4A136 group* and *Romboutsia* from the Lachnospiraceae phylum (54). This has been correlated with cancers and metabolic syndrome (55, 56) but interestingly, this was normalized after ACP mediation. ACP further alleviates NAFLD by regulating the ecological imbalance of intestinal microbiota caused by a high-fat diet.

## Conclusion

The current study showed that parameters like HOMA-IR, body fat rate, liver index, and body weight gain were significantly lowered by ACP supplementation. In addition, HDL levels were improved with a corresponding decrease in hepatic levels of TC, TG, and LDL and ALT and AST levels. ACP supplementation could alleviate oxidative stress by decreasing the hepatic levels of MDA and increasing the activities of SOD, CAT, and GSH-PX. Immunomodulatory biomarkers like IL-6, IL-1β, IL-4 and TNF-α concentrations were also significantly lowered. Interestingly, gut microbial biodiversity was restored, suggesting that this procedure could be promising in alleviating NAFLD symptoms.

## Data availability statement

The datasets presented in this study can be found in online repositories. The names of the repository/repositories and accession number(s) can be found in the article/Supplementary material.

## Ethics statement

The animal study was reviewed and approved by the Ethics Committee of the First Affiliated Hospital of Heilongjiang University of Chinese Medicine.

## Author contributions

HX and XC designed the study. WJ and YP performed the experiments. TZ wrote the manuscript. XM and JH analyzed the data. All authors contributed to the article and approved the submitted version.

## Funding

Present research work was financially supported by grants from the Natural Science Foundation of Heilongjiang Province (ZD2020C010).

## Conflict of interest

The authors declare that the research was conducted in the absence of any commercial or financial relationships that could be construed as a potential conflict of interest.

## Publisher’s note

All claims expressed in this article are solely those of the authors and do not necessarily represent those of their affiliated organizations, or those of the publisher, the editors and the reviewers. Any product that may be evaluated in this article, or claim that may be made by its manufacturer, is not guaranteed or endorsed by the publisher.

## Supplementary material

The Supplementary material for this article can be found online at: https://www.frontiersin.org/articles/10.3389/fnut.2023.1161537/full#supplementary-material
