# Supplementary material for: Assessment of Auricularia cornea var. Li. polysaccharides potential to improve hepatic, antioxidation and intestinal microecology in rats with non-alcoholic fatty liver disease
Source: Front Nutr. 2023 Jun 12;10:1161537. doi: 10.3389/fnut.2023.1161537 (PMC10292627; doi:10.3389/fnut.2023.1161537)
Supplement: Supplementary file 1 [file Table_1.DOCX]

**Table S1** Feed formula of rats

| Ingredient | Control feed (g/kg) | High fat feed (g/kg) |
| --- | --- | --- |
| Casein, 80mesh | 200 | 200 |
| L-Cystine | 3 | 3 |
| Corn starch | 315 | 0 |
| Maltodextrin 10 | 30 | 120 |
| Sucrose | 350 | 68.8 |
| Cellulose BW20 | 50 | 50 |
| Soybean oil | 25 | 25 |
| Lard | 20 | 245 |
| Mineral mix S10026 | 10 | 10 |
| Dicalcium phosphate | 12 | 12 |
| Calcium carbonate | 5.5 | 5.5 |
| Potassium citrate, 1 H_2_O | 16.5 | 16.5 |
| Vitamin mix V10001 | 10 | 10 |
| Choline bitartrate | 2 | 2 |
| FD&C yellow dye # 5 | 0.05 |  |
| FD&C blue dye # 1 |  | 0.05 |
